# Posterior atlantoaxial internal fixation using Harms technique assisted by 3D-based navigation robot for treatment of atlantoaxial instability

**DOI:** 10.1186/s12893-022-01826-2

**Published:** 2022-11-04

**Authors:** Zhao Lang, Xiaoguang Han, Mingxing Fan, Yajun Liu, Da He, Wei Tian

**Affiliations:** Department of Spine Surgery, Peking University 4th Clinical Medical College, Beijing Jishuitan Hospital, No. 31, Xinjiekou East Street, Xicheng District, Beijing, 100035 People’s Republic of China

**Keywords:** Atlantoaxial instability, Harms technique, Robot, Screw fixation, Accuracy

## Abstract

**Background:**

To evaluate the accuracy of screw placement using the TiRobot surgical robot in the Harms procedure and to assess the clinical outcomes of this technique.

**Methods:**

This retrospective study included 21 patients with atlantoaxial instability treated by posterior atlantoaxial internal fixation (Harms procedure) using the TiRobot surgical robot between March 2016 and June 2021. The precision of screw placement, perioperative parameters and clinical outcomes were recorded. Screw placement was assessed based on intraoperative guiding pin accuracy measurements on intraoperative C-arm cone-beam computed tomography (CT) images using overlay technology and the incidence of screw encroachment identified on CT images.

**Results:**

Among the 21 patients, the mean age was 44.8 years, and the causes of atlantoaxial instability were os odontoideum (n = 11), rheumatoid arthritis (n = 2), unknown pathogenesis (n = 3), and type II odontoid fracture (n = 5). A total of 82 screws were inserted with robotic assistance. From intraoperative guiding pin accuracy measurements, the average translational and angular deviations were 1.52 ± 0.35 mm (range 1.14–2.25 mm) and 2.25° ± 0.45° (range 1.73°–3.20º), respectively. Screw placement was graded as A for 80.5% of screws, B for 15.9%, and C for 3.7%. No complications related to screw misplacement were observed. After the 1-year follow-up, all patients with a neurological deficit experienced neurological improvement based on Nurick Myelopathy Scale scores, and all patients with preoperative neck pain reported improvement based on Visual Analog Scale scores.

**Conclusions:**

Posterior atlantoaxial internal fixation using the Harms technique assisted by a 3D-based navigation robot is safe, accurate, and effective for treating atlantoaxial instability.

## Background

Atlantoaxial disorders usually result in instability of the upper cervical spine and may cause spinal cord dysfunction, vascular impairment and cervical pain when left untreated for too long [1, 2]. Such disorders are mainly attributed to embryological, traumatic, or inflammatory factors. Currently, internal fixation is regarded as the main treatment for atlantoaxial instability. The Harms technique involves placement of C1 lateral mass screws in combination with C2 pedicle screws and was first described by Goel and Laheri in 1994 [3] and later by Harms and Melcher in 2001 [4]. This technique is now a popular method for atlantoaxial fixation that can achieve good pain relief and reliable biomechanical stability [5]. However, in clinical practice, screw placement has proven to be a high-risk procedure due to the complicated anatomical structures in the upper cervical region, and the possible adverse outcomes include vertebral artery and spinal cord injury. The risk is even greater when aberrant anatomy exists [6]. Relying on fluoroscopic images and surgeons’ experience alone, the conventional posterior screw insertion method is not sufficiently accurate and poses risks due to screw malposition [7].

With the rapid development of surgical robotics and its increased application in clinical practice, robotic assistance has been shown to improve the precision of screw placement in different spinal regions [8]. Compared with other surgically assisted techniques, robot-assisted spinal surgery demonstrates superior results in terms of reducing screw dislocation, shortening the duration of intraoperative radiation, and minimizing surgical bleeding [9]. Several orthopedic robotic systems, such as the TiRobot, SpineAssist, Renaissance, and Mazor, have been applied in spinal surgeries [10], and among them, the TiRobot system is the only one that can be used for posterior screw insertion in the craniocervical area [11].

In the present study, the TiRobot system was utilized to assist performance of the Harms procedure in patients with atlantoaxial instability. The resulting surgical outcomes as well as the accuracy rate of screw placement with the TiRobot surgical equipment were evaluated in the present study.

## Methods

### Patient enrollment

Patients with atlantoaxial instability due to embryological, traumatic, or inflammatory causes who underwent open or percutaneous Harms procedures in our institution between March 2016 and June 2021 were enrolled in this study. The exclusion criteria were atlantoaxial instability caused by neoplasm or infection, concurrent treatment with cervical procedures (such as anterior odontoid fixation, cervical laminoplasty or laminectomy, lower cervical pedicle screw placement, etc.), previous surgery in the upper cervical region, and failure to complete the 1-year follow-up or refusal to provide informed consent. This study was approved by the ethical board of our local institute (IRB: 201909-11).

Twenty-one patients who underwent robot-assisted Harms operation as treatment for atlantoaxial instability and met the inclusion and exclusion criteria for this study were enrolled. Among them, 11 patients were male and 10 were female. The mean patients age was 44.8 (range 19–63) years. The diagnosed causes of atlantoaxial instability in these patients included os odontoideum (n = 11, 52%), rheumatoid arthritis (n = 2, 10%), unknown pathogenesis (n = 3, 14%) and type II odontoid fracture (n = 5, 24%).

### Surgical techniques

**Fig. 1:**
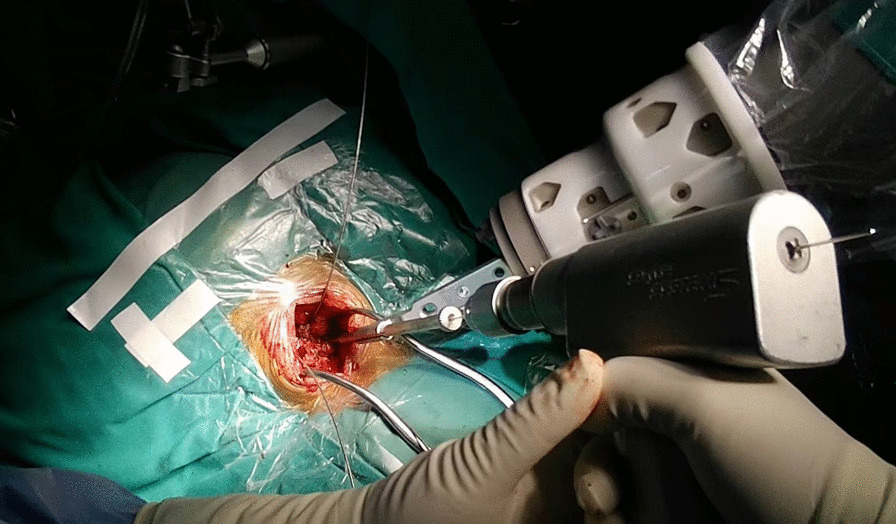
Guiding pin placement. The guiding cannula was placed onto the end of the robotic arm. Once the accuracy of guidance was less than 0.5 mm and became steady, a K-wire was drilled into the vertebrae along the guiding cannula to an optimal depth

The procedures were conducted with the TiRobot system (TINAVI Medical Technologies, Beijing, China). After general anesthesia, the patient was placed in a prone position, and the head was fixed onto the operating table with the Mayfield frame. In open surgery, the midline approach was routinely performed to expose the C1 posterior arch and C2 lamina. Then the patient tracker was anchored onto the Mayfield frame. For the percutaneous minimally invasive approach, the patient tracker was anchored first and then two para-median incision and intermuscular approaches were created under robotic guidance. The C-arm (ARCADIS Orbic 3D, Siemens Medical Solutions, Erlangen, Germany) was employed to take X-ray images, which were then transferred to the robotic workstation. The senior surgeon planned the optimal trajectory for the C1 lateral screws and C2 pedicle screws on the workstation. Then, the robotic arm was moved to the target position guided by an NDI stereo camera (Northern Digital Inc., Ontario, Canada). The guiding cannula was placed onto the end of the robotic arm. Once the deviation of guidance was within 0.5 mm and became steady, a K-wire was drilled as a guiding pin into the vertebrae along the guiding cannula to the optimal depth (Fig. 1). The position of the guiding pin was verified by re-scanning of the C-arm. Once satisfactory positioning of the guiding pin was achieved, the pilot hole was tapped, followed by insertion of 3.5-mm diameter polyaxial screws (Mountaineer, Medtronic Sofamor Danek, Memphis, TN or Summit, Depuy Spine, Raynham, MA). The cartilage of the C1–C2 articular joint was drilled out, and an autograft harvested from the iliac crest was placed into that space.

We performed the reduction maneuver by adjusting the position of the Mayfield frame and monitoring it on lateral fluoroscopy. After the reduction was satisfactory on the fluoroscopy, we acquired the 3D images using the C-arm to ensure that C1–C2 was in the anatomical reduction position or the space available for the spinal cord (distance from the posterior margin of dens to the laminar line) was more than 13 mm on the sagittal image. If the reduction procedure was satisfactory, the connecting rod and pre-tightened screw heads were installed. When needed, continuous efforts were made to adjust the atlantoaxial alignment until the reduction was satisfactory.

### Evaluation of screw placement accuracy

#### Intraoperative guiding pin accuracy

**Fig. 2:**
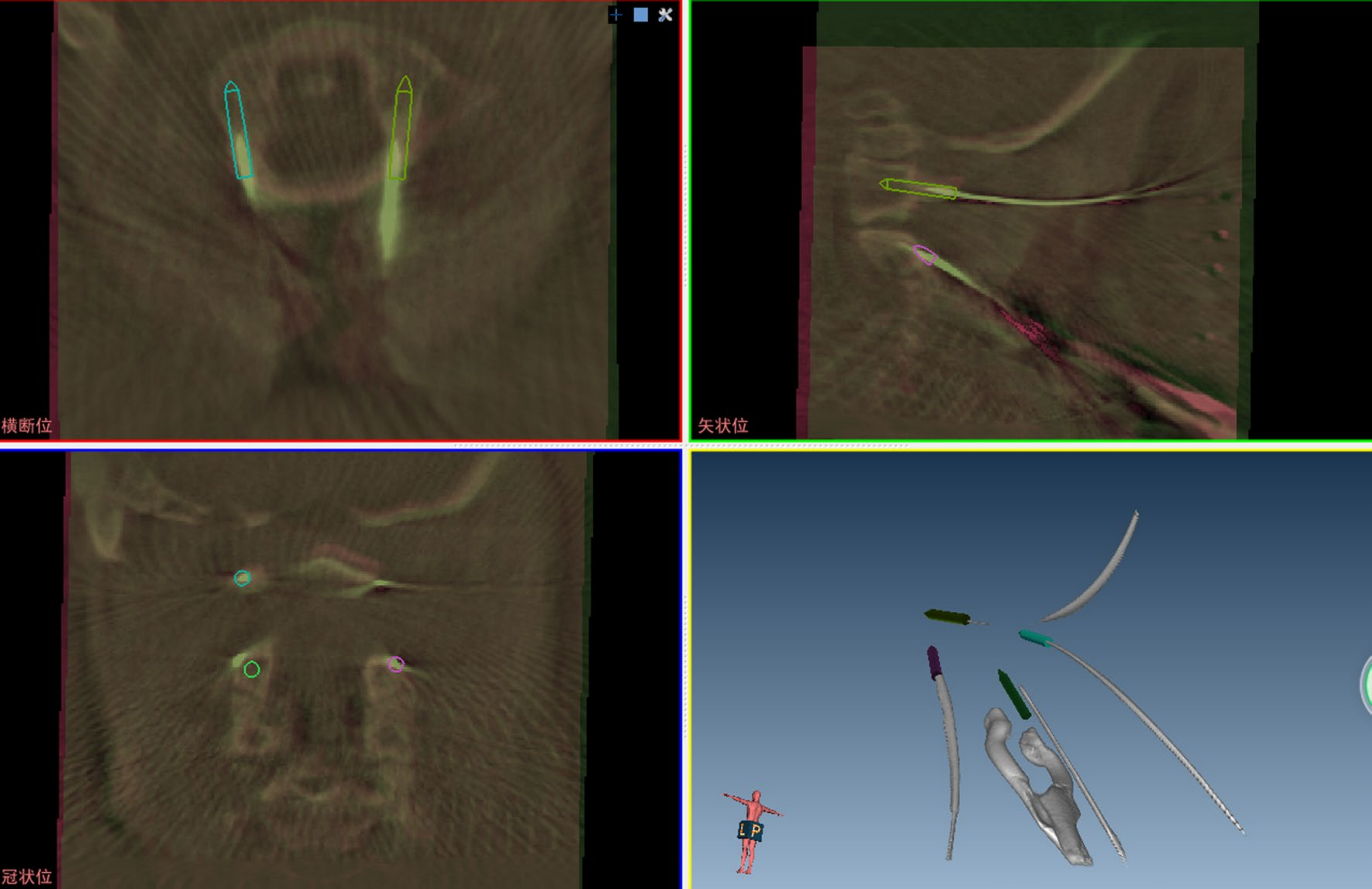
Representative fused images. Based on the manual fusion method, the screw planning image was matched with the guiding pin placement CBCT image via overlay technology in the axial plane, sagittal plane and coronal plane

Guiding pin accuracy was measured on the basis of the X-ray images obtained by the C-arm intraoperatively. The steps of the workflow were as follows:

- Step 1: Image fusion. Based on the manual fusion method through overlay technology, the screw planning C-arm cone-beam computed tomography (CBCT) image was matched with the guiding pin placement CBCT image on the TiRobot Spine Software platform (Fig. 2).
- Step 2: Entry and target point identification. The spatial coordinate values (X, Y, Z) of the entry point and target point were determined on the screw planning CBCT image as well as guiding pin placement CBCT image, separately.
- Step 3: Accuracy calculation. The accuracy of the entry point and target point were calculated separately based on Euclidean distance. Then the translational and angular deviations in the axial and sagittal planes were calculated using the following formulae:
  $$Entry\; error = \sqrt {\left( {EX - EX^\prime } \right)^{2} + \left( {EY - EY^\prime } \right)^{2} + \left( {EZ - EZ^\prime } \right)^{2} }$$
  $${\text{Target}}\; error = \sqrt {\left( {TX - TX^\prime } \right)^{2} + \left( {TY - TY^\prime } \right)^{2} + \left( {TZ - TZ^\prime } \right)^{2} }$$
  $$Total\; positioning\; error = \frac{Entry\; error + Target\; error}{2}$$

#### Screw encroachment grading

The accuracy of the screw placement also was evaluated on postoperative CT images. The precision of screw placement was classified according to three grades of the scale introduced by Gertzbein and Robbins [12]. A screw inserted completely within the pedicle was regarded as grade A; a screw inserted with a pedicle cortical breach less than 2 mm was regarded as grade B; and a screw inserted with a pedicle cortical breach exceeding 2 mm was classified as grade C.

### Clinical assessment

The operative time, estimated volume of blood loss and perioperative hospitalization duration were recorded and analyzed. Clinical examinations were performed preoperatively and 1 year after surgery and included the acquisition of Nurick Myelopathy Scale (NMS) and Visual Analog Scale (VAS) scores. All intraoperative and postoperative complications were recorded. The fusion status was assessed by CT scanning 1 year after surgery.

### Statistical analysis

All statistical analyses were performed using SPSS 26.0 (SPSS Inc, Chicago, IL). For quantitative data, differences were evaluated using the *t*-test if the data conformed to a normal distribution. *P* < 0.05 was regarded as the threshold for statistical significance.

## Results

### Operative information

Twenty of the twenty-one patients received bilateral C1 lateral screw placement together with C2 pedicle screw fixation. The only exception was a patient who had an anatomic variation that allowed for only one C1 lateral screw to be placed with one C2 pedicle screw on the left side. Thus, 82 screws were inserted in total with the aid of the robotic system. These procedures were performed by three senior spine surgeons though an open approach in 17 patients and a minimally invasive approach in 4 patients.

**Fig. 3:**
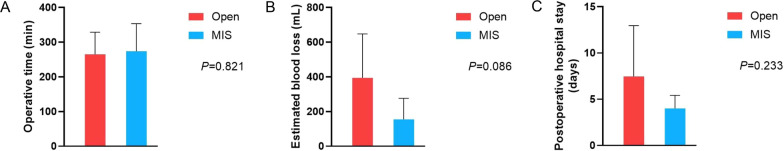
Operative parameters. Comparison of operative time (**A**), estimated blood loss (**B**) and postoperative hospital stay (**C**) between open surgery and minimally invasive surgery

Overall, the mean operative time was 266.9 ± 64.7 min, and the mean blood loss volume was 348.6 ± 250.9 mL. The mean operative time for the open procedure was 265.3 ± 63.5 min, and that for the minimally invasive procedure was 273.8 ± 79.7 min (t = − 0.0230, *P* = 0.821). The estimated blood loss volume was 394.1 ± 253.6 mL for the open procedure and 155.0 ± 121.5 mL for the minimally invasive procedure (t = 1.810, *P* = 0.086). The mean postoperative hospital stay was 6.8 ± 5.1 days for all patients, 7.5 ± 5.5 days after the open procedure and 4.0 ± 1.4 days after the minimally invasive procedure (t = 1.232, *P* = 0.233; Fig. 3).

### Screw placement accuracy

The average translational and angular deviations of the 82 screw guiding pins were 1.52 ± 0.35 mm (range 1.14–2.25 mm) and 2.25° ± 0.45° (range 1.73–3.20º), respectively. Sixty-six out of 82 screws (80.5%) were inserted with perfect precision (grade A); 13 screw placements were classified as grade B (15.9%, 13/82); and 3 were classified as grade C (3.7%, 3/82). No complications were found to be related to unsatisfactory screw placement.

### Clinical outcomes

**Fig. 4:**
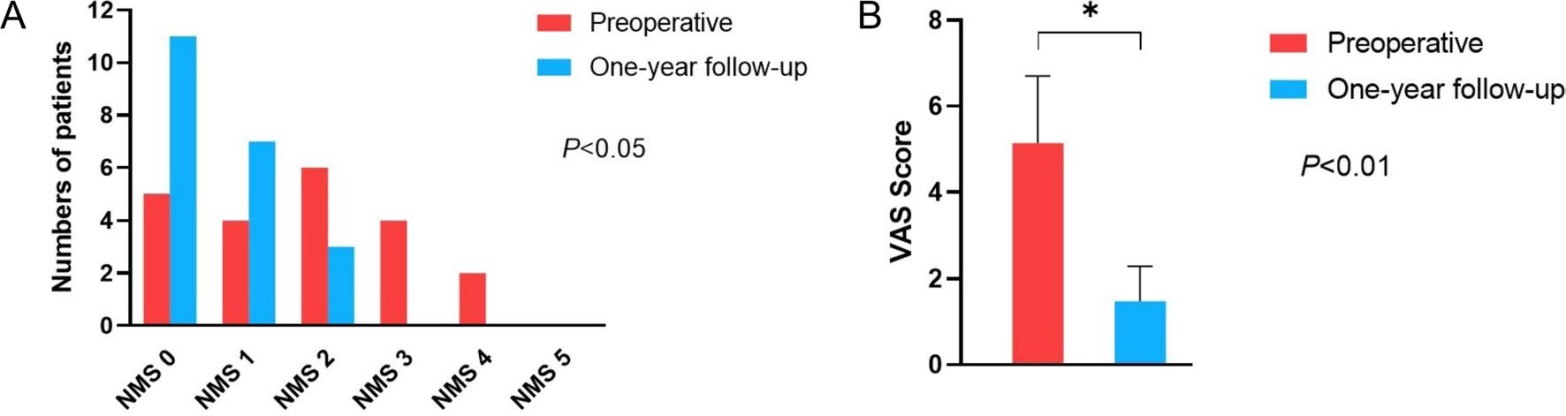
Clinical outcomes. **A** Nurick Myelopathy Scale (NMS) scores and **B** Visual Analog Scale (VAS) scores for neck pain before surgery and at 1-year follow-up

No complications were detected after 1 year of follow-up. Additionally, no additional complications were observed in percutaneous surgery, especially with regard to greater occipital neuralgia. All patients with a neurological deficit experienced neurological improvement throughout the follow-up period. The preoperative NMS scores were 0 for 5 patients, 1 for 4 patient, 2 for 6 patients, 3 for 4 patients, and 4 for 2 patients, whereas at the 1-year follow-up, 11 patients had no neurological deficits, 7 cases had a score of 1, and 3 cases had a score of 2 (Fig. 4). Symptoms were relieved or improved in all patients with preoperative neck pain, with VAS scores showing improvement by more than 70% in all of these patients (Fig. 4). All patients achieved solid fusion of the C1–C2 articular joint at 1 year postoperatively.

## Discussion

The TiRobot system is a multi-functional orthopedic surgical robotic system that can be utilized for thoracolumbar pedicle screw insertion, percutaneous vertebroplasty and lumbar-pelvic fixation [13–16].

For spinal surgery, the precision of screw placement is very important at the craniocervical junction, where the surgical window is very narrow for superior cervical screw placement, as many vital structures traverse adjacent to bony structures, making screw placement in this area dangerous. Anatomical variations make this situation even more difficult [17]. Since the Harms technique was first described, the construct has been considered safer than transarticular screws, because it is less-dependent on vertebral artery alignment due to the flexibility in screw trajectory [18, 19]. However, the risk of vertebral artery injury for C2 pars or pedicle screw placement cannot be completely avoided [20], and such injury can lead to severe blood loss, neurological impairment, stroke, and even death [21, 22].

The present study demonstrated that use of the TiRobot system could ensure the accuracy of Harms construct placement, helping to avoid complications. The high accuracy achieved with usage of the robotic system is consistent with the findings of previous reports for other types of surgeries. One meta-analysis demonstrated that the rate of acceptable pedicle screw placement, as categorized by Gerztbein-Robbin Grade A + B, was approximately 95% with the aid of the robot, which was significantly superior to the free-hand technique (odds ratio = 1.54) [23]. Compared with our present robot-assisted study, conventional methods offered much lower precision of screw placement according to the literature for Harms surgery. Zhan et al. reported that using conventional fluoroscopy guidance for Harms procedure, the proportion of “clinically acceptable” screws (graded A and B) was only 87.5% [24]. Similar results were seen in the studies conducted by Li et al. with safe screws accounting for 81.7% of 120 screws [25] and by Sancipriano et al. in which the incidence of screw malpositioning was 13% [26]. Previous studies also concluded that the application of robotics in spinal surgery greatly reduces the intraoperative time and radiation dosage in comparison to conventional procedures [23, 27]. The magnitude of screw deviations observed in the present study demonstrated good consistency with those reported previously. Van Dijk et al. used the Mazor robot to place percutaneous lumbar pedicle screws and reported a mean deviation at the entry point of 2.0 ± 1.2 mm and mean differences in the angle of insertion of 2.2° ± 1.7° on the axial plane and 2.9° ± 2.4° on the sagittal plane [28]. Devito et al. compared the planned screw insertion angles with the actual insertion of screws with the assistance of the Mazor robot and reported mean deviations of 1.2 ± 1.5 mm on the axial plane and 1.1 ± 1.2 mm on the sagittal plane [29]. A previous study by Jiang et al. showed that use of the ExcelsiusGPS surgical robot afforded a deviation of the screw tip of 2.1 mm (range 0.8–5.2 mm), a mean deviation of the screw caudal of 3.2 mm (range 0.9–5.4 mm) and a mean angular deviation of 2.4° (range 0.7–3.8°) [30]. These findings demonstrate good consistency with results in the present study, in which the TiRobot system facilitated good precision with about 1.5 mm deviation and 2° offset with an acceptable accuracy of 96.4% in the Gertzbein-Robbins evaluation. The source of this deviation is multifactorial [30, 31]. Entering the pedicle with no flat drilling surface might predispose the guiding pin to slip off the exact entry point, causing lower accuracy and higher deviation [32]. This slipping can be minimized by choosing an entry point that is not located on the steep slope of the bony surface, by pre-preparing the surface using a highspeed drill or an ultrasonic osteotome, or by using a sharp tip pin driven by a highspeed drill.

In the present study, we used two approaches, open and minimally invasive, to perform the Harms procedure. The estimated blood loss tended to be less in the minimally invasive procedure compared with the open approach, although the observed difference was not significant. Because minimally invasive screw fixation is performed through a muscle-expanding approach that significantly reduces the number of iatrogenic soft tissue injuries, it has been found to offer several potential advantages over open techniques, including reductions in blood loss, postoperative pain, recovery time, and the emotional impact on the patient [33]. Multiples studies have described minimally invasive Harms procedures [34–36]. However, a major problem of this technique is the absence of anatomical landmarks, which makes screw fixation technically challenging. The application of a navigational surgical robot is an optimal method to solve this problem. We did not compare the accuracy of screw placement between the open and minimally invasive approaches directly in the present study due to our small sample size. However, the screw accuracy overall was high with the assistance of the TiRobot system. An additional advantage of the robotic platform is that it allows the surgeon to locate the entry point at the skin level, thereby reducing the required incision size. Further studies are needed to clarify the benefit of a minimally invasive Harms technique. In the present study, the operative time and postoperative hospital stay achieved with minimally invasive surgery were comparable with those associated with the open surgery, implying the minimally invasive approach is likely as feasible as open surgery in clinical application.

The main limitation of this study was that comparison was only possible between the preoperative planning images and the observation of guiding pins on intraoperative CBCT. Although the pilot hole was tapped following placement of the guiding pin, the screw was inserted without the aid of the robot, which could lead to errors. However, because of the impact of artifacts, it is impossible to compare the preoperatively planed locations to the final screw placement on postoperative CT. This study is also limited in that it was a case series study without a control group. Thus, the benefit and utility of the robot-assisted Harms procedure remain to be elucidated in future randomized control trials. Still, the present study provides preliminarily evidence of the significance of the investigated procedure and demonstrates that promising outcomes were achieved.

## Conclusions

Posterior atlantoaxial internal fixation using the Harms technique assisted by 3D-based navigational robot is safe, accurate, and effective for the treatment of atlantoaxial instability.

## Data Availability

The datasets generated and analyzed during the current study are available from the corresponding author on reasonable request.

## Abbreviations

CBCT: C-arm cone-beam computed tomography
NMS: Nurick Myelopathy Scale
VAS: Visual Analog Scale
